# Does rabbit anti-thymocyte globulin dose and antiviral prophylaxis alter the risk of the development of PTLD in renal transplant population?

**DOI:** 10.1080/0886022X.2020.1792927

**Published:** 2020-07-14

**Authors:** Mahmoud Mohamed, Mohamed A. Ghonim, Mai M. Abouzeid, Maria Aurora Posadas Salas

**Affiliations:** Nephrology Division, Department of Medicine; University of Tennessee Health Science Center; Memphis, TN, USA; St. Jude Children’s Research Hospital, Memphis, TN, USA; Department of Immunology, Faculty of Pharmacy, Al-Azhar University, Cairo, Egypt; Louisiana State University Health Sciences Center; New Orleans, LA, USA; Division of Nephrology and Hypertension, Department of Medicine; Medical University of South Carolina; Charleston, SC, USA

Dear Editors,

We have carefully read the study published by Ali et al. on the correlation between rabbit anti-thymocyte globulin (r-ATG) and the development of post-transplant lymphoproliferative disorders (PTLD) and its aggressive forms in the renal transplant population. This is certainly a relevant study since as the authors stated; PTLD is one of the most common post-transplant malignancies. Given the impact of PTLD on mortality, it is worth investigating the factors that predispose a transplant recipient to PTLD. During an era in transplantation where the use of induction immunosuppression has become widespread, it is truly remarkable to learn the significant association of r-ATG induction therapy with PTLD, more importantly, with the aggressive types [1]. However, the authors did not correlate the incidence of PTLD with the use of antiviral prophylaxis. We cite several studies to compare and contrast with the findings of Ali et al. In a systematic literature review conducted by Marks et al. 2246 patients who underwent kidney or heart transplantation between 1999 and 2009 were included in order to assess incidence of PTLD. After a median follow-up of 5 years, they reported 0.93% incidence of PTLD among r-ATG-treated kidney recipients. . They did not find any significant association between the cumulative r-ATG dose and the risk of PTLD. In addition, patients who received antiviral prophylactic therapy with either ganciclovir, valganciclovir, acyclovir, or valacyclovir had a significantly lower incidence of PTLD compared to those who didn’t receive antiviral prophylaxis (0.63% vs. 1.87%) [2]. It would have been valuable to see the correlation of antiviral prophylaxis with the incidence of PTLD in the OPTN cohort studied by Ali et al.

Another point to comment on is that the authors did not report the cumulative dose of r-ATG administered. Laftavi et al. found better long-term renal allograft survival and lower mean serum creatinine among the patients who received low-dose r-ATG (total dose of 3–5 mg/kg) compared to those who received Basiliximab. They also observed that there was no increased risk of viral infections or cancer rates after 8 years of follow-up [3]. A study conducted on pediatric renal transplant recipients showed no significant association between r-ATG induction and the incidence of Epstein–Barr virus infection [4]. Similar findings were reported by Kute et al. [5]. Given the strong association between EBV infection and the development of PTLD, it would have been critical to investigate whether r-ATG correlated with increased incidence of EBV infection in the cohort studied by Ali et al.

In conclusion, the study conducted by Ali et al. succinctly elucidated the significant association of r-ATG induction with incidence of PTLD. Correlation of PTLD with use of antiviral prophylaxis and cumulative r-ATG dose would have been impactful as well.
